# Psychometric evaluation of the Social Touch Questionnaire in Chinese adolescents

**DOI:** 10.1002/pchj.789

**Published:** 2024-07-04

**Authors:** Chenglei Wang, Ruyun Xie, Liangliang Chen, Xinhua Yang, Chao Yan

**Affiliations:** ^1^ Shanghai Changning Mental Health Centre Shanghai China; ^2^ Key Laboratory of Brain Functional Genomics (MOE&STCSM), Affiliated Mental Health Center (ECNU), School of Psychology and Cognitive Science East China Normal University Shanghai China; ^3^ Key Laboratory of Philosophy and Social Science of Anhui Province on Adolescent, Mental Health and Crisis Intelligence Intervention Hefei China

**Keywords:** Chinese adolescents, psychometric property, social anxiety, social touch

## Abstract

Social touch promotes psychological well‐being through inducing positive effects on social affiliation and stress alleviation, especially in adolescence. However, there is a scarcity of instruments available for evaluating adolescents' attitude toward social touch in China. The present study aimed to investigate the validity and reliability of the Social Touch Questionnaire (STQ) in Chinese adolescents. A total of 2839 high school students were enrolled, ranging in age from 11 to 19 years. The dimensions of the scale were conducted by exploratory factor analysis, and validated by confirmatory factor analysis. A three‐factor model showed the best fit and consisted of the following subgroups: liking of informal social touch, liking of general social touch, and dislike of social touch. Furthermore, positive associations were found between social touch and interaction anxiety, fear of negative evaluation, depressive and anxiety symptoms, and sleep quality. Short‐term prediction of dislike of social touch in anxiety symptoms was determined, while no significant predictor of depressive symptoms was found. The STQ demonstrates reliability and validity as a self‐report questionnaire designed to assess behaviors and attitudes toward social touch. It effectively adapts to the nuances of the Chinese context among adolescents.

## INTRODUCTION

Social touch occurs in the presence of others and conveys emotional value that is stimulated and regulated by social milieus (Lima, 2022). Interpersonal touch plays an important role in receiving sensory experience, facilitating bonding, and buffering stress (Dagnino‐Subiabre, 2022; Maier et al., 2020). Lack of social connection can greatly increase one's vulnerability to stress, loneliness, anxiety, and depression (Ujitoko et al., 2022). A better understanding of social touch holds great promise for the development of early intervention techniques based on sensory characteristics, especially in adolescents (Cascio et al., 2019).

However, although social touch behaviors during childhood and adolescence are critical for healthy psychosocial development, much less research has been conducted on adolescents (Cruciani et al., 2021). Models on developmental continuity suggest that continuity from early life to adolescence and adulthood is often indirect and gradually shapes outcomes through changes in aspects of the social environment (Ulmer‐Yaniv et al., 2023). Repeated observations of the mother–child relationship over a period of 20 years reveal that the quality of maternal skin‐to‐skin contact continuously impacts maturation of human social abilities and socioemotional competencies (Ulmer Yaniv et al., 2021), which resemble the effects of alterations in maternal contact on the brains of other social animals (Liu et al., 2022). Corresponding changes in the tactile social world continue to impact brain and behavior throughout the transition to adulthood (Cascio et al., 2019). A lack of social touch in adolescence is linked to violent behaviors later in adulthood, whereas providing physical contact for adolescents with violent and conduct disorder reduces anxiety and negative emotions (Cruciani et al., 2021). Indeed, strong ties with parents, caregivers, or siblings remain supportive during this period (Orben et al., 2020). Beyond the family, adolescents tend to stay with their fellows and cultivate complex peer relationships (Foulkes et al., 2018). Compared to children or adults, adolescents have a greater sensitivity to peer acceptance, rejection, and approval. Bullying and isolation can be associated with a higher risk of developing internalizing and behavioral symptoms in adolescence (Arseneault, 2018). Mental health burden in adulthood is often the continuation of adolescent‐onset mental problems, highlighting the urgent need to comprehend why some adolescents are more prone to suffering from problems than others (Ferschmann et al., 2022; Ormel et al., 2015).

Since external social factors affect the internal mental representations of one's own body, individual psycho‐behavioral characteristics can be extracted by measuring attitude toward social touch (Martinez et al., 2022). Some self‐report questionnaires primarily focused on comfortable, positive social touch, such as the Positive Touch Experience Attitude Questionnaire (TEAQ) and the Tactile Biography (TBIO), with less attention paid to unpleasant, negative touch events (Beltran et al., 2020; Trotter et al., 2018). However, in the real world, negative affective states can be expressed via touch and closely linked to depression and anxiety (Saarinen et al., 2021; Zhu et al., 2022).

The Social Touch Questionnaire (STQ) is administered as a general measure of attitudes toward social touch, covering a wide‐ranging issue concerning positive, neutral, and negative social contact in a relatively well‐integrated manner (Wilhelm et al., 2001). The scale was originally developed to assess differences in attitudes toward touch across groups with varying levels of social anxiety and administered to a sample of female undergraduates in the United States. The original English version was later translated into Portuguese and German. The Portuguese version of the STQ contained three subscales, which were named Dislike of Physical Touch, Liking of Familiar Physical Touch, and Liking of Public Physical Touch (Vieira et al., 2016). Similarly, the German version also obtained a three‐factor model, including Dislike of Social Touch, Liking of Informal Social Touch, and Liking of General Social Touch (Lapp & Croy, 2021). The STQ has found applications across diverse health contexts. One of them is the complex association between attitudes toward touch and negative experiences in the general population: aversion toward social touch mediated the negative effect of childhood maltreatment on subjective stress (Maier et al., 2020), whereas a positive outlook on social touch instead led to more mental health sufferings, such as distress and anxiety when experiencing loneliness (Schneider et al., 2023). In clinical studies, social touch preferences of individuals with autism spectrum disorder were associated with symptom severity as well as overall affective representations and vicarious emotional sensitivity (Lee Masson et al., 2018; Lee Masson et al., 2019). Additionally, interpersonal problems in depressed patients could be partly explained by their negative attitudes toward social touch, especially aversion to physical contact with unfamiliar people (Triscoli et al., 2019). However, a major unresolved question is whether the psychometric properties exhibited by the STQ can be generalized to adolescents who are still in a period of rapid social development.

Variations in the frequency and manner of social contact arise largely from cultured rules about how to use touch in social interactions (Cascio et al., 2019; Suvilehto et al., 2019). Although the STQ has been validated in Western countries, the cross‐cultural differences in the social contexts in which Chinese adolescents develop may differentially affect some of their responses (Cascio et al., 2019). Chinese interpersonal activities are influenced mainly by Confucianism, which emphasizes the hierarchical nature of society and relationships and considers principles for social interactions, resulting in reduced body touch (Hodges & Oei, 2007). In a cross‐cultural study encompassing 45 countries, China was identified as one of the countries with the least affective touch prevalence with its more conservative societal values (Sorokowska et al., 2021). Culture also shapes sensitivity to different touch areas. Compared to Germans, Chinese reported more comfort with more public touch on the hands, and less comfort with more intimate touch on the torso and upper back (Schirmer, Cham, Zhao, Croy et al., 2022). Nevertheless, in both Western and Eastern cultures, the strength of the emotional bond is linearly associated with the permissible touch area, which implies that assessment tools developed based on other cultures could also be used to measure acceptance and avoidance toward touch behaviors in Chinese populations (Suvilehto et al., 2019), which remains to be verified.

The aim of the current study is to examine the psychometric properties of the STQ among general Chinese adolescents. In the present study, we test the following hypotheses:

**Hypothesis 1.** The STQ will have acceptable internal consistency and test–retest reliability in adolescents.

**Hypothesis 2.** There will be three factors underlying the Chinese translation of the STQ, although minor differences in composition of items may exist due to cultural differences.

**Hypothesis 3.** An association will be found between disliking social touch and anxiety.

## METHOD

### Participants

All participants were recruited from secondary schools located in the south and west of China between June and September 2022. The Southern China sample comprised of 1607 students from Guangdong province (*n* = 1005) and Guangxi province (*n* = 602), while the Western China sample comprised of 1647 students from Yunnan province. There were no significant differences among the samples in sex, age, or years of education. A total of 422 participants in the Western China sample completed the same survey with a 2‐week interval to assess test–retest reliability. Based on the following criteria, 528 participants were excluded: (1) more than 50% of data missing (*n* = 36), or failure to match with the baseline due to missing basic information (*n* = 95); and (2) the presence of prolonged sequences of invariant responses, signifying the consistent selection of the same option (*n* = 397). Specifically, participants indicating consecutive strings of more than nine invariant responses in scales (restricted to those with reverse‐scored items) were excluded (Curran, 2016). Consequently, the Southern China sample consisted of 1451 students (59.5% female), ranging in age from 11 to 18 years (*M* = 14.62 years; *SD* = 1.63 years), the Western China sample consisted of 1388 students (54.8% female), ranging in age from 13 to 19 years (*M* = 16.44 years; *SD* = 0.94 years) (Table 1), and the retest sample consisted of 309 students (62.1% female), ranging in age from 14 to 19 years (*M* = 16.61 years; *SD* = 0.98 years). Please see Table 1 and Table S1 for more details.

**TABLE 1:** Baseline demographic characteristics (*M* ± *SD*)

Variables	Western China^a^ *n* = 1388	Southern China *n* = 1451	Total^b^ *N* = 2839
Age (years)^c^	16.44 ± 0.94	14.62 ± 1.63	15.45 ± 1.63
Sex (Male/Female)	445/760	588/863	1033/1623
Scores of scales^c^			
STQ: 0–80	47.21 ± 9.05	42.70 ± 10.17	44.90 ± 9.90
BFNE: 12–60	35.43 ± 6.97		
IAS: 15–75	49.09 ± 9.08		
SDS: 25–100	56.59 ± 9.56		
SAS: 25–100	47.27 ± 9.72		
PSQI: 0–21	6.76 ± 3.09		

Abbreviations: BFNE, Brief Fear of Negative Evaluation Scale; IAS, Interaction Anxiousness Scale; PSQI, Pittsburgh Sleep Quality Index; SAS, Self‐rating Anxiety Scale; SDS, Self‐rating Depression Scale; STQ, Social Touch Questionnaire.

^a^ Samples for Western China range from 1205 to 1388 across outcomes due to missing data.

^b^ Samples for the total range from 2656 to 2839 across outcomes due to missing data.

^c^ *M* ± *SD*.

### Measurements

#### Social Touch Questionnaire

The STQ was a 4‐point Likert scale, ranging from 0 (*not at all*) to 4 (*extremely*) with a maximum total score of 80. We first obtained consent from Wilhelm before all work began. Subsequently, two experienced psychiatrists separately translated the original English version of the STQ into Chinese (forward translation). After preliminary review and revision, an initial Chinese version was independently translated back into English by two professional translators who were blinded to the original scale (backward translation). One was a psychiatrist with a doctor's degree, proficient in both Chinese and English, and another was a non‐medical Chinese American whose native language is Chinese and who worked in the United States for over 10 years. Finally, a panel of experts (consisting of one psychologist and nine psychiatrists) reviewed and confirmed the final version. Cross‐cultural adaptation was completed in this process, as well as modification to suit the age group of adolescents. For example, “kiss, cheek‐to‐cheek” was changed to “hug” in Item 14 since kissing is not a common greeting in Chinese culture; and “professor” was replaced by “teacher” in Item 8 because our target population was adolescents. No question was added or deleted. Two back‐translated versions of the questionnaire are provided in Table S2.

#### Brief Fear of Negative Evaluation Scale

The Brief Fear of Negative Evaluation (BFNE) Scale was a 12‐item concise version of the Fear of Negative Evaluation Scale, which assesses fear of negative evaluation from others, relevant to human social behavior. Rated on a 5‐point Likert scale, higher scores indicate a greater fear of negative evaluation by others (Carleton et al., 2011). The Cronbach's alpha of the scale was .746. BFNE Scale was used to evaluate convergent validity of the STQ.

#### Interaction Anxiousness Scale

The Interaction Anxiousness Scale (IAS) was a self‐reported scale regarding the affective component of social discomfort, independent of behavior, related to social anxiety (Leary & Kowalski, 1993). It comprised 15 items rated on a 5‐point scale. The Cronbach's alpha of the scale was .809. The IAS was used to evaluate convergent validity of the STQ.

#### Self‐rating Depression Scale

The Self‐rating Depression Scale (SDS) was used to measure the degree of depression, including 20 items, scored on a 4‐point Likert scale (Zung, 1965). After multiplying the total score by 1.25, the integer part was taken as the standard score (25–100). Participants who scored ≥53 were identified as exhibiting depressive symptoms (Jiang et al., 2022). The reviewed literature indicates that highly depressed individuals have more negative attitudes toward social touch compared to those with minimal to mild depression (Triscoli et al., 2019). The SDS was used to evaluate concurrent and predictive validity of the STQ. The Cronbach's alpha of the scale was .774.

#### Self‐rating Anxiety Scale

The Self‐rating Anxiety Scale (SAS) was developed to evaluate the frequency of anxiety. It was composed of 20 items rated on a 4‐point Likert scale (Zung, 1971). Like the SDS, the standard score was the integer part of the total score multiplied by 1.25. Anxious individuals often experience relational difficulties that cause significant distress, and are more likely to avoid social touch (Passarelli et al., 2022). Consequently, the SAS was used to evaluate concurrent and predictive validity of the STQ. The Cronbach's alpha of the scale was .814.

#### Pittsburgh Sleep Quality Index

The Pittsburgh Sleep Quality Index (PSQI) was designed for quantitative and qualitative sleep assessment (Buysse et al., 1989). Its Chinese version was composed of 19 questions divided into seven components, with higher scores implying worse sleep quality. Previous studies have demonstrated that changes in children's sleep quality are not strongly linked to the extent of their social contact (Mühlematter et al., 2024). The PSQI was therefore used to evaluate divergent validity of the STQ. The Cronbach's alpha of the scale was .711.

### Procedure

The Southern China sample participated on WJX (wjx.com), a platform providing functions equivalent to Amazon Mechanical Turk. Permission was obtained by sending an informed consent to guardians online via the school–parent app. The survey took approximately 20 min to complete. A total of 422 of these participants were randomly invited to complete the same survey 2 weeks later. The Western China sample was conducted in groups of 30–50 individuals sitting in classrooms, with the help of their teachers. Informed consent from guardians was obtained via the school–parent app as well. On average, participants took 20–30 min to complete the paper‐and‐pencil survey during a regularly scheduled class period. Students were not individually compensated for participation but were given small incentives (e.g., sweets).

### Data analysis

IBM‐SPSS Version 22 (IBM Corp., Armonk, NY) and Mplus Version 8.3 (Muthén & Muthén, 1998–2017) were used for the analyses. The total sample was split randomly into Samples A and B. We conducted exploratory factor analysis (EFA) using Sample A (*n* = 1384, 713 from Southern China and 671 from Western China), while Sample B (*n* = 1455, 738 from Southern China and 717 from Western China) was utilized for confirmatory factor analysis (CFA) to validate the identified factor structure derived from the EFA. The Kaiser–Meyer–Olkin (KMO) test (>0.7) and Bartlett's test of sphericity (*p* < .05) were conducted to review adaptability at first. Robust model fit indices for the CFA included the following: robust chi‐square/degrees of freedom (<5 indicates acceptable); comparative fit index (CFI), and Tucker–Lewis index (TLI, >.90, acceptable); standardized root‐mean‐square residual (SRMR), and root‐mean‐square error of approximation (RMSEA, <.08, acceptable). Validity of the individual model factors was assessed through the average variance extracted (AVE) and construct reliability (CR) in CFA. Spearman correlation analyses were carried out to evaluate concurrent, convergent, and divergent validity between the STQ and other scales in the Western China sample. And stepwise multiple regression analysis was used to explore the predictive validity as the dependent variable and demographic factors as baseline predictor variables. The internal consistency was assessed using Cronbach's alpha, and test–retest reliability was calculated using the intraclass correlation (ICC) at a 2‐week interval from the Western China sample. The *t*‐test for independent samples was performed for evidence of sex differences. Bonferroni‐adjusted *p*‐values were used to control the family‐wise error rate when making multiple comparisons.

## RESULTS

### Exploratory factor analysis

**FIGURE 1:**
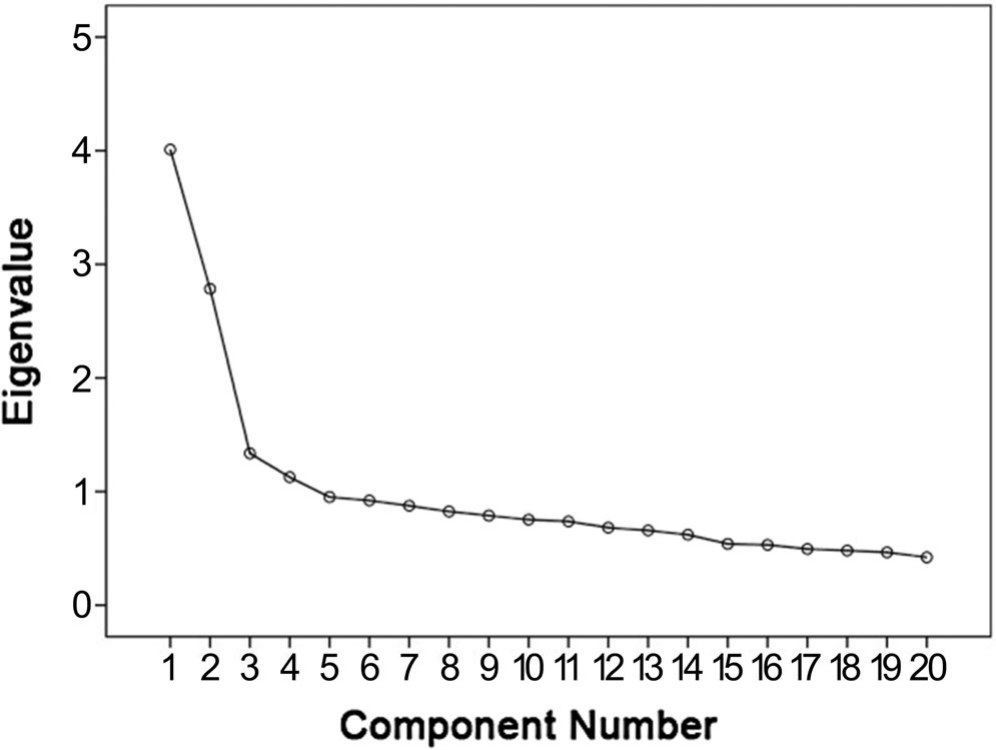
Scree plot of exploratory factor analysis (EFA) for Chinese version of the Social Touch Questionnaire. EFA suggested four factors with Eigenvalues higher than one.

Statistically significant *p*‐values (*p* < .001) in Bartlett's test, along with a KMO measure of sampling adequacy at 0.85, indicated the eligibility of the factor analysis. Consistent with the three‐factor model proposed by Lapp & Croy (2021) and Vieira et al. (2016), a three‐component solution was yielded based on the scree plot (Figure 1). The eigenvalues of the first three factors were 4.0, 1.3, and 2.8. Further inspection of the factor scores explained 19.4% of the total variance on the first factor, 9.5% on the second factor, and 11.8% on the third factor (see Table 2).

**TABLE 2:** Factor loadings of each item in a three‐factor model.

Items	F1	F2	F3
Factor 1: Dislike of Social Touch			
10. I feel uncomfortable if I make physical contact with a stranger on the bus or subway.	**0.708**	−0.094	−0.008
7. It annoys me when someone touches me unexpectedly.	**0.687**	0.131	−0.148
17. It would make me feel anxious if someone I had just met touched me on the wrist.	**0.684**	0.087	−0.155
3. I get nervous when an acquaintance keeps holding my hand after a handshake.	**0.674**	−0.112	−0.046
8. I'd feel uncomfortable if a teacher^a^ touched me on the shoulder in public.	**0.656**	0.188	−0.156
13. I would rather avoid shaking hands with strangers.	**0.605**	−0.249	0.020
2. I feel uncomfortable when someone I don't know very well hugs me.	**0.587**	−0.105	0.158
5. I feel embarrassed if I have to touch someone in order to get their attention.	**0.548**	−0.092	0.039
16. I feel disgusted when I see public displays of intimate affection.	**0.532**	0.116	−0.055
19. I hate being tickled.	**0.461**	−0.133	0.009
Factor 2: Liking of Informal Social Touch			
9. I'd be happy to give a pat on shoulder or a hug^a^ to a friend if they are feeling stressed.	−0.047	**0.674**	0.205
12. As a child, I was often cuddled by family members (e.g., parents, siblings).	−0.029	**0.632**	−0.088
14. I greet my close friends with a hug.^a^	−0.043	**0.571**	0.318
20. I like petting animals.	−0.048	**0.503**	0.118
Factor 3: Liking of General Social Touch			
4. I generally seek physical contact with others.	0.024	0.066	**0.783**
1. I generally like when people express their affection towards me in a physical way.	−0.017	0.247	**0.693**
11. I like close physical contact^a^ in intimate situations (e.g., hug, kiss).^b^	0.046	0.277	**0.677**
6. I consider myself to be a “touchy‐feely” person.	−0.044	0.327	**0.479**
18. If I had the means, I would get weekly professional massages (e.g., *Tui Na*).^b^	−0.023	0.001	**0.456**
15. I feel comfortable touching people I do not know very well.	−0.173	−0.140	**0.324**

*Note*: Bold values indicate the factor attribution and corresponding factor loading for each item, enhancing readability.

^a^ Means a modification in words.

^b^ Means an additional supplement for understanding.

### Confirmatory factor analysis

**FIGURE 2:**
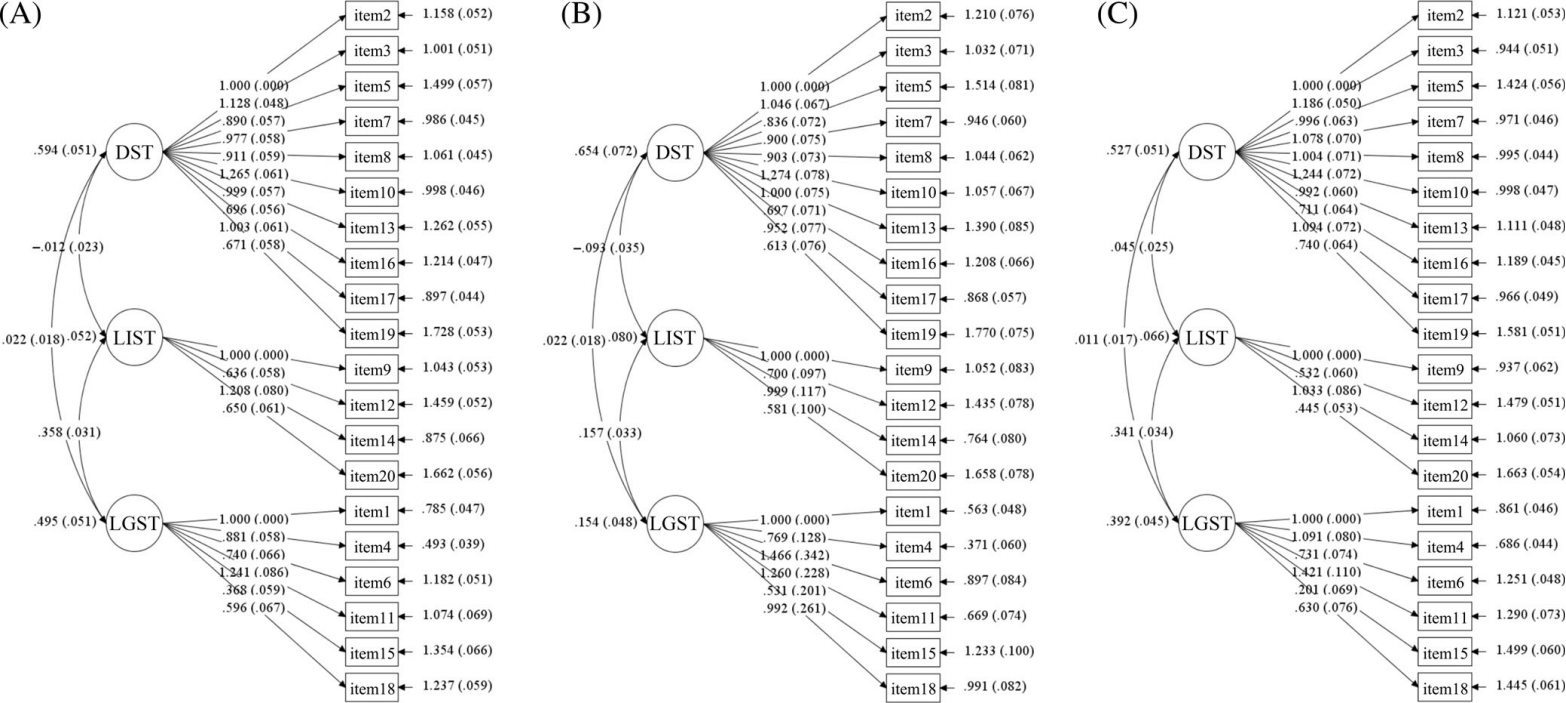
Path diagram for the confirmatory factor analysis of (A) the overall sample, (B) the Western China sample, and (C) the Southern China sample. The confirmatory factor analysis was carried out separately for the Southern China sample (*n* = 738), the Western China sample (*n* = 717) and the total groups (*n* = 1455), based on a three‐factor structural model, using the maximum likelihood robust estimator. The fitted indicators of the validated factors were basically within the reference range, and the models were acceptable. DST, Dislike of Social Touch; LIST, Liking of Informal Social Touch; LGST, Liking of General Social Touch.

Confirmatory factor analysis confirmed the three‐factor structure (i.e., Dislike of Social Touch, Liking of Informal Social Touch, and Liking of General Social Touch). The overall model was found to be acceptable, with *χ* ^2^/*df* (628.656/167) = 3.764, RMSEA = .044, CFI = .903, TLI = .889, and SRMR = .044. A subsequent analysis also found evidence that the Southern China and Western China samples demonstrated good fit (Southern China: *χ* ^2^/*df* (790.856/167) = 4.736, RMSEA = .051, CFI = .864, TLI = .846, and SRMR = .045; Western China: *χ* ^2^/*df* (340.315/167) = 2.038, RMSEA = .038, CFI = .912, TLI = .900, and SRMR = .045; see Table 3 and Figure 2). In addition, the subclinical depression group, categorized using a cutoff score of 53 or above on the SDS, was utilized for factor analysis. Similarly, a three‐component solution was selected and validated. Further inspection of the factor composition revealed that the subclinical depression model was identical to the overall model in the items comprising each factor. The results are presented in Tables S3–S5.

**TABLE 3:** CFA goodness of fit indices of the overall model.

Indices	Total (*n* = 1455)	Western China (*n* = 717)	Southern China (*n* = 738)
*χ* ^2^/*df*	3.764	2.038	4.736
RMSEA	0.044	0.038	0.051
CFI	0.903	0.912	0.864
TLI	0.889	0.900	0.846
SRMR	0.044	0.045	0.045

	DST	LIST	LGST	DST	LIST	LGST	DST	LIST	LGST
AVE	0.316	0.277	0.266	0.325	0.255	0.186	0.326	0.255	0.225
CR	0.815	0.586	0.661	0.823	0.560	0.560	0.825	0.549	0.593
Cronbach's α	0.818	0.579	0.637	0.819	0.545	0.531	0.822	0.536	0.560

Abbreviations: AVE, average variance extracted; CFA, Confirmatory Factor Analysis; CFI, comparative fit index; CR, construct reliability; DST, Dislike of Social Touch; LGST, Liking of General Social Touch; LIST, Liking of Informal Social Touch; RMSEA, root‐mean‐square error of approximation; SRMR, standardized root‐mean‐square residual; TLI, Tucker–Lewis index.

### Convergent and divergent validity

As shown in Table S3, positive correlations were found between the STQ and interaction anxiety (*r* = 0.488, *p* < .001), as well as fear of negative evaluation (*r* = 0.239, *p* < .001), suggesting that the scale had a good convergent validity. Subscale analyses revealed that Dislike of Social Touch exhibited positive associations with interaction anxiety (*r* = 0.535, *p* < .001) and fear of negative evaluation (*r* = 0.317, *p* < .001), whereas Liking of Informal Social Touch and Liking of General Social Touch did not show any significant correlations. In addition, there was a mild association between the STQ and sleep quality (*r =* 0.139, *p* < .001), suggesting good divergent validity. The STQ was significantly associated with age (*r* = 0.118, *p* < .001). Sex difference existed, with females reporting more discomfort about social touch than males (*t* (2334.205) = 12.884, 95% CI = 4.118 to 5.597, *p* < .001).

### Concurrent and predictive validity

Significant associations between the STQ and depressive symptoms (*r* = 0.214, *p* < .001) as well as anxiety symptoms (*r* = 0.147, *p* < .001) were found, suggesting adequate concurrent validity (see Table S6). Moreover, Dislike of Social Touch was positively associated with the severity of depression (*r* = 0.185, *p* < .001) and anxiety (*r* = 0.225, *p* < .001), while Liking of General Social Touch was only negatively associated with anxiety symptoms (*r* = −0.123, *p* < .001). Additionally, a small correlation emerged between depressive symptoms and Liking of Informal Social Touch (*r* = 0.092, *p* = .001). In terms of predictive validity, after controlling the influence of age and sex ratio (*R* ^2^ = 0.017, adjusted *R* ^2^ = 0.010, *p* = .036), only Dislike of Social Touch predicted the changes of anxiety symptoms after 2 weeks, explaining an additional 1.7% of the variance (*R* ^2^ = 0.034, adjusted *R* ^2^ = 0.024, *p* = .021; *β* = 0.180, *p* = .026). However, the STQ and its factors did not exhibit a significant predictive effect on depressive symptoms (see Table 4).

**TABLE 4:** The results of multilevel regression analysis.

Measure	Model	Adjusted *R* ^**2**^	*B*	*SE*	*β*	*t*
Depression symptoms						
STQ	Unadjusted	0.017	0.149	0.061	0.143	2.436*
	+ age, gender	0.028	0.111	0.063	0.107	1.753
DST	Unadjusted	0.016	0.153	0.070	0.128	2.180*
	+ age, gender	0.034	0.103	0.073	0.086	1.399
LIST	Unadjusted	−0.003	0.061	0.195	0.019	0.314
	+ age, gender	0.017	0.008	0.194	0.002	0.041
LGST	Unadjusted	0.002	0.216	0.176	0.073	1.226
	+ age, gender	0.024	0.253	0.174	0.085	1.453
Anxiety symptoms						
STQ	Unadjusted	0.016	0.157	0.067	0.138	2.344*
	+ age, gender	0.018	0.127	0.070	0.112	1.823
DST	Unadjusted	0.023	0.214	0.076	0.164	2.801**
	+ age, gender	0.024	0.180	0.081	0.138	2.238*
LIST	Unadjusted	0.001	−0.248	0.214	−0.069	−1.163
	+ age, gender	0.013	−0.298	0.214	−0.083	−1.395
LGST	Unadjusted	−0.001	0.154	0.193	0.047	0.799
	+ age, gender	0.009	0.187	0.193	0.057	0.968

*Note*: STQ, DST, LIST, LGST = Social Touch Questionnaire and its three factors (Dislike of Social Touch, Liking of Informal Social Touch, and Liking of General Social Touch); Depressive symptoms = Self‐rating Depression Scale (SDS); Anxiety symptoms = Self‐rating Anxiety Scale (SAS).

* *p* < .05;

** *p* < .01.

### Internal consistency and test–retest reliability

Samples A and B were merged to calculate the internal consistency. The Cronbach's alpha of the total scores and three factors were .707, .818, .564, and .638, respectively. The intraclass correlation coefficient was .764 (*p* < .001), and for the three subscales, it stood at .719, .467, and .624 (*p* < .001), indicating acceptable test–retest reliability.

## DISCUSSION

This study examined the psychometric characteristics of the STQ in a substantial cohort of Chinese adolescents. The STQ exhibited high internal consistency and good validity, as evidenced by the coefficients of Cronbach's alpha and Spearman's correlation. The Chinese translation version of the scale also appeared to be characterized by three factors, which were in line with the earlier findings in college student populations (Lapp & Croy, 2021; Vieira et al., 2016).

Consistent with expectations, the three‐component model derived from factor analysis reveals a robust bimodal pattern centered around disliking (Factor 1) and liking (Factor 2 and 3). As noted in the German study, liking included informal social touch (Factor 2) and general social touch (Factor 3) (Lapp & Croy, 2021). The former mainly concerned the tendency toward affective behaviors in casual situations, whereas the latter involved more abstract concepts of touching attitudes, including self‐perception and willingness for social proactivity, suggesting an attitudinal divergence between treating abstract and concrete situations. According to the dual theoretical model of neural network for abstract and concrete cognition, while concrete concepts pertain to perceptual information discernable from past experiences, abstract concepts involve insights transcending the immediate environment (Gotlieb et al., 2022; Liberman & Trope, 2008; Motofei & Rowland, 2013). Participants' tendencies to produce concrete and abstract explanations were unrelated (Gotlieb et al., 2022), and measuring each aspect separately might reflect independent socio‐cognitive traits, theoretically supporting the result that liking was divided into two factors. The findings of the factor structure indicate that the scale had adequate construct validity to assess attitude toward social touch in a multidimensional manner.

CFA revealed that the items related to dislike were stable and highly concentrated, while the items that constituted the two liking factors varied somewhat due to cultural differences (Lapp & Croy, 2021; Vieira et al., 2016). For example, Item 11 deals with close body contact, involving hugs and kisses, which are common in Western countries but generally limited to intimate relationships in Eastern Asia (Suvilehto et al., 2019), making this question an abstract scenario for Chinese adolescents. In addition, differences in the sample populations might also have contributed to these inconsistencies (e.g., Item 9, “I'd be happy to give a pat on shoulder or a hug to a friend if they are feeling stressed”) (Beltran et al., 2020). Students who spend considerable time with their peers may find more opportunities to express comfort through a pat on shoulder or a hug, making Item 9 a more concrete scenario. These findings need further evaluation and validation in a larger and more diverse adolescent sample from Eastern cultures as well as those from Western cultures.

Correlation analysis found that the STQ had moderate association with interaction anxiety, fear of negative evaluation, depressive and anxiety symptoms, providing some evidence of convergent and concurrent validity for the scale in adolescents. Consistent with prior studies, individuals with higher scores of STQ displayed more deficits in fear of negative evaluation and anxiety, indicating the relationship between aversion linked to body contact and social anxiety (Birk et al., 2019; Chiu et al., 2021). Further subscale analyses revealed that the terms “liking” and “dislike” target different constructs and do not form a continuum. The Dislike of Social Touch subscale primarily includes items related to incidental or passive touch, whereas the Liking of Informal Social Touch and Liking of General Social Touch subscales refer mainly to pleasant affective touch. Given that social anxiety is characterized by an elevated intolerance of uncertainty (Clauss et al., 2019), the lack of control in situations involving interpersonal touch may be particularly uncomfortable for individuals with high interaction anxiety and fear of negative evaluation. Conversely, it can be speculated that social situations that are comfortable and controllable may provoke less anxiety. In addition, subscale analysis indicated individuals experiencing depression or anxiety share a similar attitude toward social touch: a higher level of depression or anxiety is often linked to a greater aversion to touch with strangers, consistent with previous research findings (Elias & Abdus‐Saboor, 2022; Triscoli et al., 2019). However, divergence exists over the attitudes toward pleasant affective touch: individuals with depressive symptoms tend to exhibit a similarly reduced tendency toward intimate touch in casual situations (Hensel et al., 2023), whereas anxiety symptoms may prompt a desire for affective touch provided by others, reflecting a potential willingness to seek comfort and support (Fine et al., 2018). Moreover, the STQ demonstrates acceptable divergent validity, although it did have a low but significant correlation with sleep quality. Regression analysis found that compared to the two liking factors, Dislike of Social Touch positively predicted the change in anxiety symptoms after 2 weeks. Likewise, no significant predictor of depressive symptoms was found either. Similar results have been observed in previous longitudinal studies that childhood social problems predicted anxiety disorders in adulthood but not depressive disorder (Roza et al., 2003). Although individuals with depression often report interpersonal problems, structural equational modeling revealed that social skills do not have an influence on depression (Cole & Milstead, 1989). Moreover, dislike of social touch could point to fear and rejection of social behaviors and result in anxiety symptoms later, since anxiety was regarded as more in line with a “fear” presentation than depression (Cummings et al., 2014). The difference in the predictive role of social problems attested to a stable link between social problems and anxiety that did not decrease over time, and different developmental pathways for anxiety and depression due to diverse roots in childhood (Roza et al., 2003). Combined with cognitive models of social anxiety (Taylor & Wald, 2003), it is reasonable to hypothesize that distinct relationships exist between social–cognitive measures and social anxiety, distinguished from general anxiety and depression.

The present study showed good internal consistency, whereas test–retest reliability was not excellent but at an acceptable level, consistent with prior studies (Lapp & Croy, 2021; Vieira et al., 2016; Wilhelm et al., 2001). Similar to the problem encountered by our German colleagues, Liking of Informal Social Touch exhibits moderate reliability. Concrete individualized experiences might be overly sensitive to minor perturbations, which contributed to the reduced stability compared to the other two factors (Bechlivanidis et al., 2017; Lapp & Croy, 2021). Additionally, the model initially recommended by the EFA included more than three factors, and artificial compression might affect both subscales referring to preference for social touch. An enlarged STQ based on Chinese culture deserves future consideration.

Furthermore, we found that the scale was influenced by age and sex. Previous research revealed a propensity of increasing age making individuals more positive toward touch in general (Sehlstedt et al., 2016). The preference for affective touch stimuli appears to grow as age increases, which might contribute to a significant relation between attitudes toward social touch and age (Cruciani et al., 2021). Consistent with the German version, females in the Chinese sample reported more discomfort about social touch than males. This may be due to the fact that females are more likely to have affectively negative descriptions about their experiences as touch receivers than males (Schirmer, Cham, Zhao, Croy et al., 2022; Schirmer, Cham, Zhao, Lai et al., 2022).

As far as we know, the present research was the first to assess the eligibility of the STQ in adolescents. However, several limitations of the current work should be addressed in further studies. First, most study participants were from the south and west of China, and the results might differ from region to region. Second, this study was limited to a community sample. The generalizability of our findings needs to be further tested in clinical samples, and further examination is required to determine whether the scale is useful for distinguishing patients with varying psychopathological disorders. Third, additional longitudinal studies over a longer period of time will also be needed to assess whether measurement invariance of the STQ would be stable across time, and how well the scale can predict anxiety symptoms. Finally, considering that the EFA initially recommended a model that included more than three factors, there is room for improvement of the STQ. More in‐depth research on an enlarged scale should follow.

Findings from this study highlight the universal importance of social touch in adolescent development across different cultures. As a fundamental form of communication, social touch not only fosters connections and intimacy among individuals, but also serves as an essential component in the development of emotional health. When adolescents are undergoing significant physiological and psychological changes, social touch plays a crucial role in shaping their identity, self‐esteem, and social skills. Although China has been characterized as having lower touch frequency and a more conservative social attitude when compared to Western cultures, such as those in North America and Europe (Schirmer, Cham, Zhao, Croy et al., 2022; Schirmer, Cham, Zhao, Lai et al., 2022), results from this study suggest that many of the factors underlying aversion and preference toward social touch may be comparable. In both Europe and China, it appears that self‐reported attitudes toward social touch center around dislike of social touch, liking of informal social touch, and liking of general social touch. The ability to accurately assess social touch preferences and aversions among adolescents holds significant potential in early identification of those exhibiting social withdrawal tendencies. Social withdrawal, often manifesting as avoidance of social interactions and touch, can be a precursor to more severe emotional and behavioral issues, such as depression, anxiety, and autism spectrum disorders. By identifying these individuals early on, researchers and practitioners can implement targeted prevention strategies and intervention programs aimed at encouraging positive social touch and building social connections. The present study supported the use of the STQ as an easy, simple‐to‐administer, reliable and valid questionnaire for assessing social touch in adolescents, and paved the way for future studies that could further explore the therapeutic potential of social touch in promoting emotional health and well‐being.

## CONFLICT OF INTEREST STATEMENT

The authors have no competing interests to declare that are relevant to the content of this article.

## ETHICS STATEMENT

All procedures performed in studies involving human participants were in accordance with the ethical standards of Shanghai Changning Mental Health Center, the East China Normal University and with the 1964 Helsinki Declaration and its later amendments or comparable ethical standards. The study was approved by the Ethics Committee of Shanghai Changning Mental Health Center (M202009) and The Ethics Committee of the East China Normal University (HR 257‐2021).

## Supporting information


**Data S1.** Supporting information.
