# Supplementary material for: Psychometric evaluation of the Social Touch Questionnaire in Chinese adolescents
Source: Psych J. 2024 Jul 4;13(6):943–53. doi: 10.1002/pchj.789 (PMC11608799; doi:10.1002/pchj.789)
Supplement: Supplementary file 1 — Data S1. Supporting information. [file PCHJ-13-943-s001.docx]

**Supplementary Materials**

**Table S1** demographic characteristics of the second wave **(N=309)**

|  | **followup^b^  N=309** | |
| --- | --- | --- |
| **Age** (years)**^a^** | 16.61±0.98 | |
| **Sex** (Male/Female) | 95/192 | |
| **Scores of scales ^a^** |  |  |
| STQ: 0-80 | 48.07±9.51 | |
| BFNE: 12-60 | 34.87±6.99 | |
| IAS: 15-75 | 46.84±9.00 | |
| SDS: 25-100 | 57.19±9.40 | |
| SAS: 25-100 | 48.04±10.19 | |
| PSQI: 0-21 | 6.53±3.31 | |
| Note: STQ = Social Touch Questionnaire; BFNE = Brief Fear of Negative Evaluation scale; IAS = Interaction Anxiousness Scale; SDS = Self-Rating Depression Scale; SAS = Self-Rating Anxiety Scale; PSQI = Pittsburgh sleep quality index. | | |
| a：Mean ± Standard deviation; b：samples range from 287 to 309 across outcomes due to missing data. | | |

**Table S2** Two back-translated versions of the questionnaire

|  | | **Back translation（1st）**by a psychiatrist with doctor degree and an overseas study tour more than a year in America |
| --- | --- | --- |
| Please choose the best answer to the following statement that meets your characteristics or real situation  0=Not at all, 1=Slightly agree, 2=Generally agree, 3=Very agree, 4=totally agree | | |
| 1 | | I usually like people to express their feelings for me in body language. |
| 2 | | I feel uncomfortable when the unacquaintance embraces me. |
| 3 | | I feel nervous when someone I know but is not familiar enough to hold my hand after shaking hands with me. |
| 4 | | I usually seek physical contact with others. |
| 5 | | I will feel embarrassed if I have to touch someone to get their attention. |
| 6 | | I consider myself a person with "exposed emotions". |
| 7 | | I am very angry when someone bumps into me by accident. |
| 8 | | I feel uncomfortable when the professor pat on my shoulder in public. |
| 9 | | If my friends feel stressed, I will be willing to do a neck/shoulder massage for them. |
| 10 | | If I have physical contact with strangers on the bus or subway, I will feel uncomfortable. |
| 11 | | I like be caressed in intimate situations. |
| 12 | | When I was young, I was often hugged by my family (for example, my parents, brothers and sisters). |
| 13 | | I would rather avoid shaking hands with strangers. |
| 14 | | I greet my intimate friends with a cheek-to-cheek kiss. |
| 15 | | I feel comfortable when I touch people I don’t know much about. |
| 16 | | I feel disgusted when seeing someone showing intimacy in public. |
| 17 | | If a new acquaintance touches my wrist, it will make me feel anxious. |
| 18 | | If there’s a way, I will receive professional massage every week. |
| 19 | | I hate being tickled. |
| 20 | | I like petting animals. |
|  | |  |
|  | **Back translation（2nd）**by a Chinese American living in New York and having a non-medical job | |
| Item | |  |
| Introduction: Please pick out the option that fits your characteristics or real condition.  0=Not agree at all, 1=Slightly agree, 2=Generally agree, 3=Very agree, 4=Fully agree | | |
| 1 | | I normally like people to express their feelings about me through body language. |
| 2 | | I feel uncomfortable when a person who I don't know hugs me. |
| 3 | | I feel nervous when someone I know but don’t familiar with, holds my hands after shaking hands with me. |
| 4 | | I usually seek physical contact with others. |
| 5 | | If I had to touch someone to get their attention, I would feel embarrassed. |
| 6 | | I consider myself a person who tends to show affection in public. |
| 7 | | I will be very angry when someone bumped into me by accident. |
| 8 | | I will feel uncomfortable if the professor touches my shoulder in public. |
| 9 | | I would like to do a neck/shoulder massage for my friends who are under stress. |
| 10 | | If I had physical contact with strangers on the bus or subway, I would feel uncomfortable. |
| 11 | | I like to be caress on intimate occasions. |
| 12 | | When I was young, I was often hugged by my family (for example my parents, brothers, and sisters). |
| 13 | | I would rather not shake hands with people I don’t know. |
| 14 | | I say hello to my close friends via a cheek-to-cheek kiss. |
| 15 | | I feel uncomfortable when I touch a person who I don’t know much about. |
| 16 | | I feel sick when I see people display intimacy in public. |
| 17 | | I feel anxious when someone I just met touches my wrist. |
| 18 | | If I have resource, I will receive a professional massage every week. |
| 19 | | I hate being tickled |
| 20 | | I am fond of touching animals. |

**Table S3** General profiles of adolescents in the Subclinical depression group and the Healthy group

|  | **Subclinical depression^b^ N=900** | **Healthy^c^ N=488** | ***χ2/t*** | ***p*** |
| --- | --- | --- | --- | --- |
| **Age** (years)**^a^** | 16.41±0.93 | 16.49±0.97 | -1.45 | .15 |
| **Sex** (Male/Female) | 242/537 | 203/223 | 32.53 | .000** |
| **Scores of STQ^a^** |  |  |  |  |
| Total score: 0-80 | 48.12±8.88 | 45.53±9.12 | 5.15 | .000** |
| DST subscore: 0-40 | 18.94±8.21 | 17.21±7.69 | 3.89 | .000** |
| LIST subscore: 0-16 | 8.93±3.19 | 8.23±3.36 | 3.84 | .000** |
| LGST subscore: 0-24 | 20.26±3.15 | 20.09±3.10 | 0.97 | .33 |

Note: STQ, DST, LIST, LGST= Social Touch Questionnaire and its three factors (Dislike of social touch, Liking of informal social touch, and Liking of general social touch);

a Mean ± Standard deviation.

b samples for the Subclinical depression group range from 779 to 900 across outcomes due to missing data.

c samples for the Healthy group range from 426 to 488 across outcomes due to missing data.

**Table S4** Factor Loadings for each Item in the Subclinical depression model

| Items | F1 | F2 | F3 |
| --- | --- | --- | --- |
| *Factor 1: Dislike of social touch* |  |  |  |
| 10.I feel uncomfortable if I make physical contact with a stranger on the bus or subway. | **.744** | -.032 | .030 |
| 7.It annoys me when someone touches me unexpectedly. | **.653** | -.104 | -.022 |
| 17.It would make me feel anxious if someone I had just met touched me on the wrist. | **.724** | -.085 | -.122 |
| 3.I get nervous when an acquaintance keeps holding my hand after a handshake. | **.662** | .053 | .076 |
| 8.I’d feel uncomfortable if a **teacher^*^** touched me on the shoulder in public. | **.633** | -.037 | -.040 |
| 13.I would rather avoid shaking hands with strangers. | **.527** | -.337 | -.010 |
| 2.I feel uncomfortable when someone don’t know very well hugs me. | **.650** | -.036 | .052 |
| 5.I feel embarrassed if I have to touch someone in order to get their attention. | **.555** | .070 | -.038 |
| 16.I feel disgusted when I see public displays of intimate affection. | **.538** | -.082 | -.116 |
| 19.I hate being tickled. | **.472** | -.168 | .076 |
| *Factor 2: Liking of informal social touch* |  |  |  |
| 9.I’d be happy to give **a pat on shoulder or a hug^*^** to a friend if they are feeling stressed. | -.169 | **.618** | .083 |
| 12.As a child, I was often cuddled by family members (e.g. parents, siblings). | .097 | **.742** | .025 |
| 14.I greet my close friends with **a hug^*^**. | -.087 | **.671** | .141 |
| 20.I like petting animals. | -.118 | **.358** | .290 |
| *Factor 3: Liking of general social touch* |  |  |  |
| 4.I generally seek physical contact with others. | .127 | -.063 | **.665** |
| 1.I generally like when people express their affection towards me in a physical way. | -.012 | .105 | **.656** |
| 11.I like **close physical contact^*^** in intimate situations **(e.g. hug, kiss)^**^**. | .057 | .030 | **.680** |
| 6.I consider myself to be a ‘touchy-feely’ person. | -.097 | .128 | **.494** |
| 18.If I had the means, I would get weekly professional massages **(e.g. Tui Na)^**^**. | .047 | .155 | **.531** |
| 15.I feel comfortable touching people do not know very well. | -.206 | .012 | **.263** |

^*^ Means a modification in words.

^**^ Means an additional supplement for understanding.

**Table S5** CFA fit indices for the Subclinical depression model

|  | Subclinical Depression  （n=451） | | |
| --- | --- | --- | --- |
| Chi^2^/df | 1.908 | | |
| RMSEA | 0.045 | | |
| CFI | 0.878 | | |
| TLI | 0.861 | | |
| SRMR | 0.058 | | |
|  | **DST** | **LIST** | **LGST** |
| AVE | 0.334 | 0.220 | 0.165 |
| CR | 0.830 | 0.497 | 0.510 |
| Cronbach’s α | 0.829 | 0.485 | 0.457 |

Note: CFA= Confirmatory Factor Analysis; RMSEA= Root Mean Square Error of Approximation; CFI= Comparative Fit Index; TLI= Tucker-Lewis Index; SRMR= Standardized Root Mean Square Residual; AVE= Average Variance Extracted; CR= Construct Reliability; DST= Dislike of social touch; LIST= Liking of informal social touch; LGST= Liking of general social touch.

**Table S6** The Spearman correlation coefficients between the STQ and other scales in the West China sample

|  | **STQ** | **DST** | **LIST** | **LGST** |
| --- | --- | --- | --- | --- |
| Interaction anxiety | 0.488* | 0.535* | -0.059 | 0.051 |
| Fear of negative evaluation | 0.239* | 0.317* | -0.066 | -0.082* |
| Sleep quality | 0.139* | 0.192* | -0.016 | -0.090* |
| Depressive symptoms | 0.214* | 0.185* | 0.092* | 0.035 |
| Anxiety symptoms | 0.147* | 0.225* | -0.033 | -0.123* |

Note: STQ, DST, LIST, LGST= Social Touch Questionnaire and its three factors (Dislike of social touch, Liking of informal social touch, and Liking of general social touch); Interaction anxiety= Interaction Anxiousness Scale (IAS); Fear of negative evaluation= Brief Fear of Negative Evaluation scale (BFNE); Sleep quality= Pittsburgh sleep quality index (PSQI); Depressive symptoms= Self-Rating Depression Scale (SDS); Anxiety symptoms= Self-Rating Anxiety Scale (SAS). Bonferroni correction: **p*< .0025.
